# Self-Oscillation-Based Frequency Tracking for the Drive and Detection of Resonance Magnetometers

**DOI:** 10.3390/s16050744

**Published:** 2016-05-21

**Authors:** Zheng Tian, Dahai Ren, Zheng You

**Affiliations:** Department of Precision Instruments, Tsinghua University, Beijing 100084, China; tz13@mails.tsinghua.edu.cn

**Keywords:** MEMS, magnetometer, self-oscillation, temperature drift, resonance sensor

## Abstract

This paper reports a drive and detection method for Micro-Electro-Mechanical System (MEMS)-based Lorentz-force resonance magnetometers. Based on the proposed MEMS magnetometer, a drive and detection method was developed by using self-oscillation to adjust the mismatch between the mechanical resonance frequency and the coil drive frequency as affected by temperature fluctuations and vibration amplitude changes. Not only was the signal-to-noise ratio enhanced by the proposed method compared to the traditional method, but the test system automatically reached resonance frequency very rapidly when powered on. Moreover, the linearity and the measurement range were improved by the magnetic feedback generated by the coil. Test results indicated that the sensitivity of the proposed magnetometer is 59.6 mV/μT and its noise level is 0.25 μT. When operating in ±65 μT, its nonlinearity is 2.5‰—only one-tenth of the former prototype. Its power consumption is only about 250 mW and its size is only 28 mm × 28 mm × 10 mm, or about one-eighth of the original sensor; further, unlike the former device, it can distinguish both positive and negative magnetic fields. The proposed method can also be applied in other MEMS sensors such as gyroscopes and micromirrors to enhance their frequency tracking ability.

## 1. Introduction

Magnetometers are widely used in navigation, attitude determination, mining, and other applications. Aerocrafts such as satellites are often required to maintain a certain attitude when operating in outer space. By sensing the magnetic field by using a magnetometer, the attitude of the satellite can be calculated by comparing the measurement result with the standard model of the geomagnetic field. Many magnetometers have been researched extensively, including flux gate magnetometers, magnetoresistance magnetometers, Hall-effect magnetometers, and atomic magnetometers. Compared to Micro-Electro-Mechanical System (MEMS) resonance magnetometers, flux gate magnetometers are larger in size and consume more power. Magnetoresistance magnetometers have the disadvantage of hysteresis. Hall-effect magnetometers are relatively sensitive and can be easily affected by the temperature. Although they have extremely high resolution, atomic magnetometers are limited to a certain extent by the environment in which they must operate. Resonance magnetometers offer a reasonable alternative for aerocraft attitude determination systems.

The first study on resonance magnetometers was published in 1991 by Donzier and Lefort, who built a cantilever structure magnetometer with a coil on its surface [1]. The cantilever vibrates when the coil is driven by the current, while the sensor detects the piezoresistance at the root of the cantilever. In 1997, Eyre and Kdr reported two torsional-structure magnetometers with a piezoresistor and capacitor as detectors, respectively [2,3]. After that, new readout methods were proposed like optical and frequency [4,5]. In 2008, Kyynäräinen improved the coil structure into a spiral, improving the sensitivity compared to the traditional coil [6]. In 2012, Li proposed a three-axis Lorentz-force magnetic sensor which exploits the in-plane and out-of-plane movement to acquire the values of a three-axis magnetic field [7]. In 2015, Minott achieved ultra-low consumption by combining the MEMS sensor with the AISC circuit [8]. Li proposed a novel quadrature-frequency modulation method to improve the bandwidth of the sensor [9], and Sonmezoglu presented force-rebalanced operation with enhanced scale-factor and bandwidth [10]. In 2016, Lara‑Castro improved sensor resolution and reduced circuit size compared to previous models via a new generating algorithm [11]. The two biggest bottlenecks of the Lorentz-force magnetic sensor currently are temperature drift of mechanical resonance frequency and bandwidth. In recent years, the research trends focus on performance optimization [12,13,14,15,16,17], integrating with inertial device [18] and discovering new readout method [19].

We previously developed a magnetometer that achieved a sensitivity of 30 nT [20]. Unfortunately, the mismatch between the drive frequency and the resonance frequency hampered its practical application. There are mainly two methods used in frequency tracking: self-oscillation [21,22,23,24,25] and phase locked loop (PLL) [26,27,28,29]. Here, we present a solution to this problem based on a closed-loop control which realizes drive frequency tracked with mechanical resonance frequency. A comparison among the key parameters of existing sensors is shown in Table 1.

## 2. Resonance Magnetometer Based on Lorentz-Force

### 2.1. Working Principle and Fabrication

**Figure 1:**
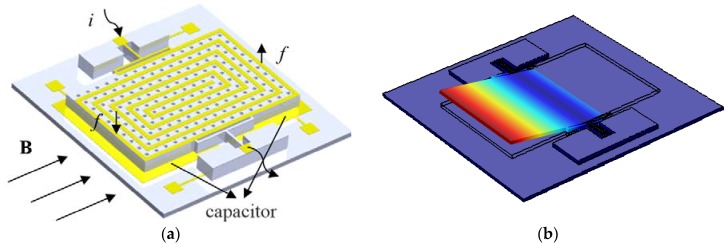
(**a**) Schematic diagram of magnetometer; (**b**) operation vibration mode via FEM simulation.

The structure of the MEMS Lorentz-force resonance magnetometer is shown in Figure 1. The size of the entire device is 4.4 mm × 4.3 mm. As shown in Figure 1a, a current *i* with the frequency at the MEMS structure resonance point was loaded to the spiral coil. As the pendulum (with holes) swings around the beams driven by the Lorentz-force, as shown in Figure 1b, the differential capacitors between the pendulum and two gold plates below it provide the change in capacitance to obtain the magnetic field intensity along direction B.

**Figure 2:**
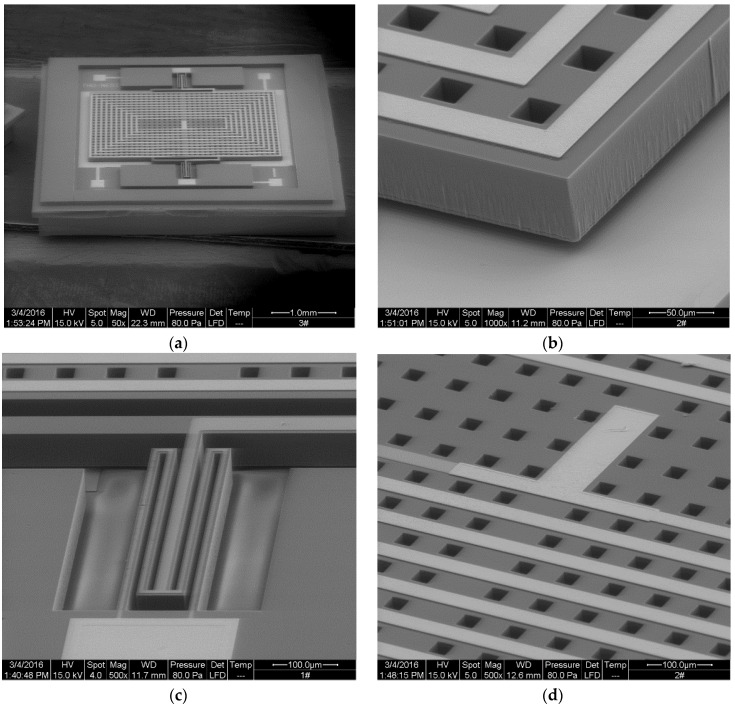
(**a**) MEMS structure of the device; (**b**) partial enlargement of the gap between pendulum and lower plate; (**c**) partial enlargement of the torsion beam; (**d**) partial enlargement of the pendulum.

A full view and partial SEM images of the MEMS structure are shown in Figure 2. We designed two types of structures with different beam widths, the main structural parameters of which are listed in Table 2. The MEMS magnetometer structure has high sensitivity when working at high *Q* value, which is determined by the air damping. The tests results showed that the *Q* value of the second-order system is about 1.5 when working in air, while about 300 in a 30 Pa vacuum. Squeeze film damping was the main effect of low *Q* value. The eight magnetometers we tested all have different mechanical resonance frequencies ranging from 957 Hz to 993 Hz.

When the magnetometer works, a sine voltage (with amplitude of about 0.15 V) is loaded to the coil with the resistance ranging from 341 Ω to 383 Ω, therefore, the current in the coil is about 0.4 mA. To improve the signal-to-noise ratio, we used a 1 MHz and ±5 V sine voltage for modulation in the middle plate. The upper plates were made of low-resistivity silicon with resistance of 150 Ω; this low resistance can reduce the crosstalk between the driving sine signal and the lower detection plate. Since the capacitance between the upper and the lower plates is about 3 pF, the max change in capacitance was about ±0.6 fF when the external magnetic field density was 50 μT.

### 2.2. Frequency Tracking Principle Based on Self-Oscillation

**Figure 3:**
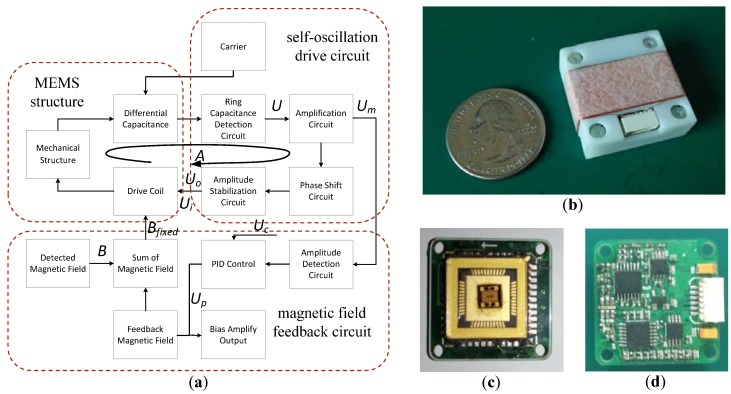
(**a**) Block model of the closed-loop control; (**b**) the prototype; (**c**) packaged MEMS structure; (**d**) circuit of the closed-loop control.

The closed-loop drive was developed based on self-oscillation. The block model (Figure 3a) contains three parts: the MEMS structure, self-oscillation drive circuit, and magnetic field feedback circuit. The self-oscillation drive circuit consists of a ring capacitance detection circuit, an amplification circuit, a phase shift circuit, and an amplitude stabilization circuit, where the ring capacitance detection circuit serves to demodulate the vibration signal from the modulated signal. To keep the MEMS structure oscillating and to obtain a linear output, we designed a feedback coil to generate a feedback magnetic field. The feedback coil drive contains an amplitude detection circuit and a PID control circuit. The output of the PID control circuit was measured to determine the intensity of the magnetic field.

The self-oscillation drive circuit is similar to a Wien bridge circuit. It contains two parts: one for frequency selection and one for amplification. The frequency selection part consists of a MEMS structure and a phase shift circuit, taking advantage of the second-order system features. When working at its resonance frequency, the phase of the vibration is 90° behind the driving signal. When the *Q* value is high, its phase is sensitive to the drive frequency. When the self-oscillation drive circuit works, the closed-loop phase shift meets the following condition:

(1) $$\theta_{MEMS}+\theta_{\text{circuit}}=2n\pi (n=0,\pm 1,\pm 2\cdots )$$

where *θ_MEMS_* is the phase shift of the MEMS structure and *θ_circuit_* is the phase of the circuit. The amplitude meets:

(2) B⋅A=1

where *B* is the density of the external magnetic field and *A* is the transformation coefficient from drive voltage *U_i_* to the output voltage of the amplitude stabilization circuit *U_o_*, which can be obtained from Equations (3)–(9). Equation (3) shows the relationship between *U_i_* and *I*, where *I* is coil current and *R*_0_ is coil resistance:

(3) $$I=\frac{U_{i}}{R_{0}}$$

The torque of the pendulum *M* can be obtained as follows:

(4) M=BILl

where *B* is the density of the external magnetic field, *L* is the length of the coil, and *l* is the equivalent arm of the Lorentz-force. The angle of the deflection *φ* can be obtained from Equation (5) when the air damping can be neglected:

(5) $$\varphi =Q\frac{M}{k}$$

where *Q* is the quality factor and *k* is the spring constant. Because the angle is small, the change in capacitance can be obtained as follows:

(6) ΔC=cφ

where *c* is the scale coefficient.

The output of the ring capacitance detection circuit *U* is:

(7) $$U=\frac{2(V-V_{D})\Delta C}{C_{0}}$$

where *V* is the amplitude of carrier, *V_D_* is the forward voltage of the rectifier diode, and *C*_0_ is the initial capacitance. *U* is amplified *F* times to obtain *U_o_*:

(8) $$U_{o}=FU$$

where *F* is the amplification factor of amplification, phase shift, and amplitude stabilization circuit.

As *U_o_* feeds back to drive the coil of the MEMS sensor, *U_i_* is equal to *U_o_*. Based on the above formulas, parameter *A* can be obtained as follows:

(9) $$A=Ll\cdot \frac{Q}{k}\cdot c\cdot \frac{2(V-V_{D})}{C_{0}}\cdot \frac{F}{R_{0}}$$

Importantly, *A* is nonlinear due to the nonlinearity of the rectifier diode and the amplifier, making every *B* correspond to output voltage *U_m_* of the amplification circuit. Although an amplitude stabilization circuit should be designed to produce nonlinear characteristics, it was omitted here because there were nonlinear characteristics in the amplification circuit and the phase shift circuit.

**Figure 4:**
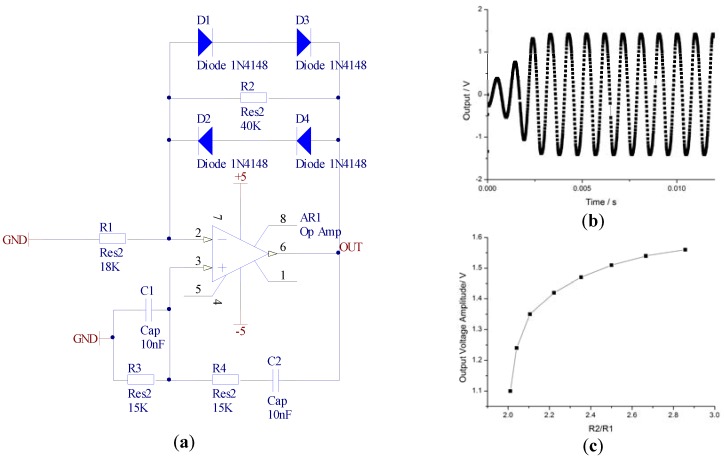
(**a**) Simulation model of the Wien bridge circuit; (**b**) simulation output of the OUT point; (**c**) relationship between R2/R1 and output voltage.

The self-oscillation system can be simplified as a Wien bridge circuit, which is a type of sine-wave generator. The simulation model is shown in Figure 4a. The ratio of R2/R1 can be seen as an external magnetic field density. The output of OUT has different voltages when R2/R1 varies; the nonlinear characteristics are provided by diodes D1, D2, D3, and D4. Figure 4b is the simulation result of OUT, where the stable amplitude is about 1.42 V when R1 is 18 KΩ and R2 is 40 KΩ. Figure 4c demonstrates that the output voltage amplitude changes from 1.1 V to 1.55 V while R2/R1 ranges from 2.01 to 2.86, which effectually explains the working principle of the self-oscillation system.

The magnetic field feedback circuit provides an additional magnetic field which reflects the output of the self-oscillating drive circuit and provides the kernel self-oscillating drive circuit with a stable, fixed magnetic field. The output voltage of the PID control circuit can be expressed as follows:

(10) $$aU_{p}+B=B_{fixed}$$

(11) $$U_{p}=\frac{1}{a}B_{fixed}-\frac{1}{a}B$$

where *a* is the coefficient from the output voltage *U_p_* to the feedback magnetic field, and *B_fixed_* is the sum of the external and feedback magnetic fields, which is fixed and related to the sensitivity of the self-oscillation drive circuit from *B* to amplitude of *U_m_* and control voltage *U_c_*.

The final prototype is shown in Figure 3b. The prototype is 28 mm × 28 mm × 10 mm in size, and consists of a shell made with a 3D printer, a feedback copper coil, a MEMS magnetometer sensor packaged in a LCC44 package (Figure 3c), and a drive and detection circuit (Figure 3d).

## 3. Testing the Magnetometer

**Figure 5:**
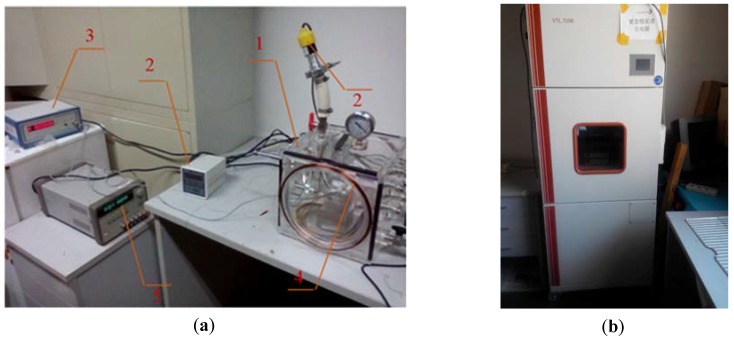
Magnetometer testing. (**a**) Test platform: (1) PMMA vacuum chamber; (2) vacuometer; (3) magnetometer; (4) Helmholtz coil; (5) power source; (**b**) Temperature and climatic chamber for the temperature experiment.

A photo of the test platform is shown in Figure 5. The magnetometer prototype was placed in the center of a polymethyl methacrylate (PMMA) vacuum chamber. 

A resistance gauge tube vacuometer was used to monitor the air pressure and a magnetic flux gate magnetometer was employed to calibrate the external magnetic field density produced by a Helmholtz coil and the geomagnetic field. Test voltage signal generation, data acquisition, and data processing were achieved via a NI myDAQ DAQ and a Labview virtual instrument program. The temperature experiment was conducted in a VTL 7006 temperature and climatic test chamber (Voetsch, Balingen-Frommern, Germany).

## 4. Experimental Results

### 4.1. Features of the Open-Loop Prototype

**Figure 6:**
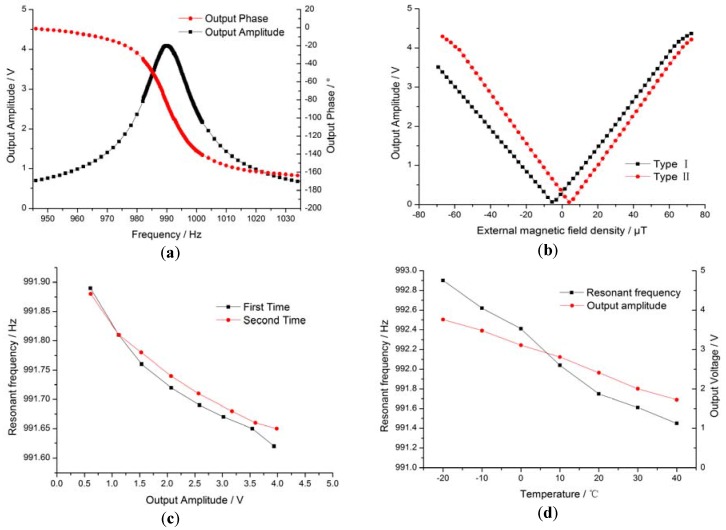
Features of the open-loop prototype. (**a**) Bode diagram of open-loop prototype; (**b**) sensitivity of the open-loop prototype; (**c**) vibration amplitude drift of the resonance frequency; (**d**) temperature drift of the resonance frequency and amplitude.

Prior to the experiment, the external magnetic field density was calibrated. We first placed the open-loop prototype in the center of the vacuum chamber and used the vacuum pump to generate a high vacuum, then loaded a 0.15 V sine signal to drive the spiral coil. The features of the resulting open-loop prototype are shown in Figure 6. Figure 6a shows an open-loop Bode diagram of the MEMS structure and the detection circuit, illustrating the features of the MEMS resonant structure. The resonance frequency of the resonant structure was 990 Hz with an 84.9° phase lag. As the *Q* value increased from 1.5 in the air to 39.6 in the vacuum environment, the phase sensitivity was about −7°/Hz at the resonance frequency. Figure 6b shows the magnetic field sensitivities of the open-loop prototype, which were 57 mV/μT and 64 mV/μT, respectively. The zero points of the two prototypes drifted from the zero magnetic fields to 4.4 μT and −6.0 μT, likely due to the magnetic field brought by the Kovar alloy pins of the package and the placement direction of the prototype. Saturation was identified when output voltage was 4 V; nonlinearity at −4 V and 4 V was 5% and 12%, respectively.

The resonant frequency of the MEMS torsional pendulum changes when the vibration amplitude or the temperature changes. The resonance frequency changes caused by the vibration amplitude drift and the temperature drift are shown in Figure 6c,d. In Figure 6c, the output voltage changed with the external magnetic field, reflecting the vibration amplitude, which was about 0.2 Hz at the output voltage of 4 V. The resonance frequency decreased by 0.3 Hz with the vibration amplitude, most likely due to the drift of the interior stress when there were torsion deformations in the rectangular beam; this was not observed in the bending deformation. Similar characteristics can be found in frequency-detection-type magnetic sensors [37]. Figure 6d shows the temperature drift of the resonance frequency and the amplitude from −20 °C to 40 °C, where the resonance frequency decreased by 1.5 Hz (0.025 Hz/°C) and the output voltage decreased by 54% (9000 ppm). There were two reasons for the decrease in the resonance frequency: the temperature drift in Young's modulus and the change in beam size. Resonance frequency *ω_r_* can be expressed as follows [38]:

(12) $$\omega_{r}=\sqrt{(1-2\alpha^{2})\frac{k}{\Theta }}$$

(13) $$k=\frac{Ehw^{3}}{2\left(1+\mu \right)d}[\frac{1}{3}-\frac{64w}{\pi^{5}h}\sum_{n=1,3,5...\infty }^{\infty }\left(\tanh\frac{n\pi h}{2w}/n^{5}\right)]$$

(14) $$\Theta =\frac{\rho DhW^{3}}{24}$$

where *α* is the damping ratio, *k* is spring constant, *Θ* is half moment of inertia, *E* is Elastic modulus, *μ* is Poisson ratio, *h* is beam thickness, *w* is beam width, *d* is beam length, *ρ* is silicon density, and *W* and *D* are the length and width of the pendulum. The temperature drift of *E* is a major factor [39]. The resonance frequency drift made it difficult to obtain stable sensitivity with the fixed coil drive signal, especially at high *Q* values.

### 4.2. Features of Self-Oscillation Closed-Loop

**Figure 7:**
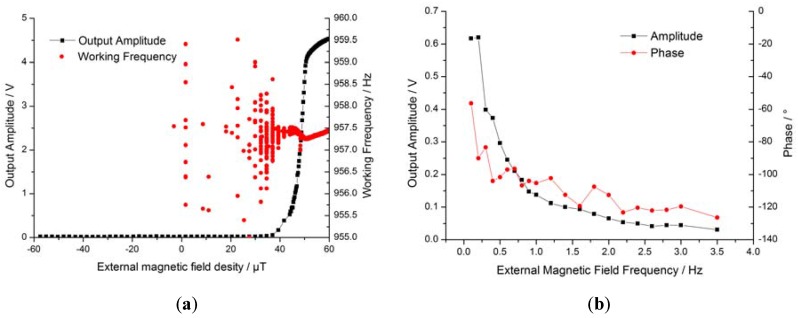

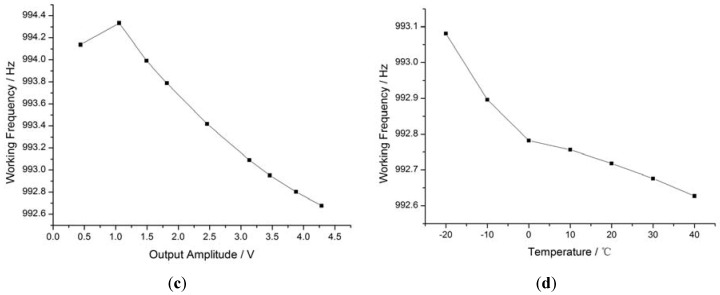
Features of the self-oscillating closed-loop system. (**a**) Sensitivity of closed-loop prototype; (**b**) response bandwidth; (**c**) vibration amplitude drift of the working frequency; (**d**) temperature drift of the working frequency.

The self-oscillating closed-loop circuit is another approach to driving the coil to track the resonance frequency. The features of the circuit are shown in Figure 7. The relationship between the external magnetic field density and the output voltage is shown in Figure 7a. In contrast with the open-loop prototype, the closed-loop driving method can only allow a positive sensing direction, which makes it possible to distinguish positive and negative magnetic fields as well as the sensitivity characteristic changes in the full range. The range was divided into three parts accordingly: a non-sensitive zone, a high-sensitivity zone, and a saturated zone. The maximum sensitivity was 1.00 V/μT in the high-sensitivity zone, which was about 20 times than that of the open-loop method. 

The red points in Figure 7a indicate the working frequency at each external magnetic field test point, where the stability of the working frequency increases while resonance amplitude increases. The average working frequency was about 957.3 Hz, almost equal to the mechanical resonance frequency (957.6 Hz). Among the test results for all eight prototypes, deviations between the working frequency and the mechanical resonance frequency were consistently below 0.3 Hz. Figure 7b shows a Bode diagram of the closed-loop prototype, where the bandwidth of the resonance magnetometer is only a few Hertz. The frequency tracking effect of the closed-loop circuit is shown in Figure 7c,d. The vibration amplitude drift of the working frequency was about 1.6 Hz (clearly larger than 0.3 Hz) and the temperature drift of the working frequency was about 0.4 Hz (*i.e.*, less than 1.5 Hz). The two charts show that the self-oscillation closed-loop circuit performs frequency tracking well, although the frequency tracking effect is not quite sufficient due to the fact that the phase of the circuit also changes with the amplitude and the temperature drift. Further, working frequency is the coupling result determined by Equation (1); its partial differentiation Equation (15) shows the key factors of frequency tracking:

(15) $$\frac{\partial \theta_{MEMS}}{\partial \omega }\Delta \omega +\frac{\partial \theta_{\text{circuit}}}{\partial \omega }\Delta \omega +\frac{\partial \theta_{\text{circuit}}}{\partial T}\Delta T+\frac{\partial \theta_{\text{circuit}}}{\partial U}\Delta U=0$$

where *ω*, *T*, *U* are working frequency, temperature, and amplitude, respectively.

Under ideal conditions, *θ_circuit_* should equal 90°. The smaller the coefficient of the last three parts in practice the better, as this ensures the smallest ∆*ω* while ∂*θ_MEMS_*/∂*ω* should be as large as possible; that is to say, frequency tracking effect is better when the sensor works in a higher vacuum.

**Figure 8:**
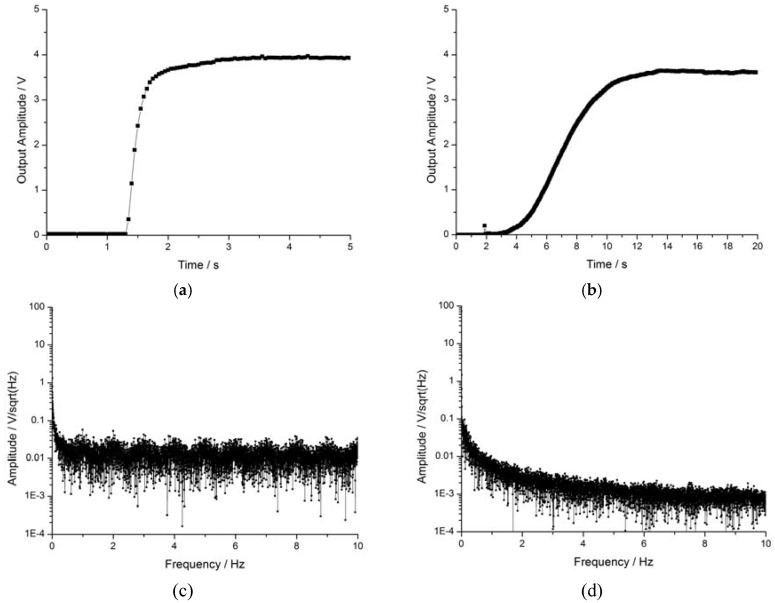
Comparison between two kinds of drive methods. (**a**) Rise time of open-loop prototype; (**b**) rise time of self-oscillating closed-loop prototype; (**c**) noise spectrum of open-loop prototype; (**d**) noise spectrum of self-oscillating closed-loop prototype.

As shown in Figure 8a,b, the rise time of the open-loop prototype was about 1.7 s but was 11.5 s in the self-oscillating closed-loop prototype, *i.e.*, almost 6.7 times that of the former. Despite the relatively long rise time, the closed-loop prototype is advantaged by its ability to automatically seek the resonance frequency. Clearly, the noise level of the self-oscillating closed-loop prototype was lower than the open-loop prototype as shown in Figure 8c,d. The noise of the open-loop prototype was about 25 mV/√Hz, while that of the self-oscillating closed-loop prototype was about 2 mV/√Hz. In effect, the self-oscillation closed-loop driving method leads to reduced noise. The frequency-selection characteristic of self-oscillation not only enhances the frequency near the resonance frequency, but suppresses the remaining frequency; additionally, the higher of the *Q* value the better the effect.

### 4.3. Features of the Closed-Loopcoil Feedback Prototype

**Figure 9:**
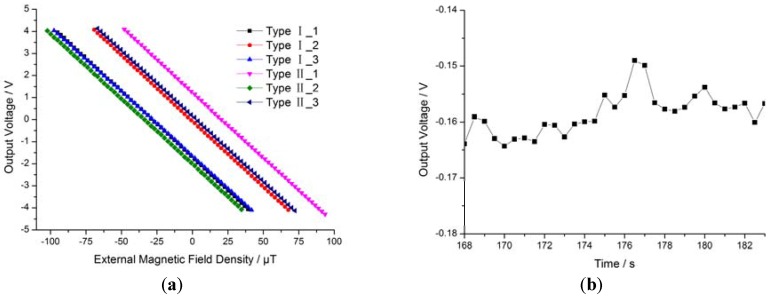
Features of the closed-loop coil feedback system. (**a**) Sensitivity of the closed-loop coil feedback prototype; (**b**) noise.

Based on the self-oscillation closed-loop control method discussed above, we developed a closed-loop coil feedback model that can solve two problems: the vibration problem around the zero magnetic field and the nonlinearity problem of the self-oscillating closed-loop drive circuit. The output characteristics of the proposed system are shown in Figure 9. Figure 9a shows the sensitivity of six closed-loop coil feedback prototypes of two types (three of each), which is about 59.6 mV/μT. The sensitivity is determined by the feedback coil parameters, making it readily adjustable. The zero point varied in a wide range, from −2.10 V to +1.14 V, as primarily related to the magnetic field carried in the package and the bias circuit. 

This suggests that the zero point consistency can be improved by reducing the magnetic field carried in the package or by adjusting the bias circuit parameters. The operating range covers the geomagnetic field of about ±65 μT, and the nonlinearity is 2.5‰—both better than the open-loop prototype. Figure 9b shows the final noise of the sensor, about 15 mV (equivalent to 0.25 μT).

## 5. Conclusions

We have developed a novel drive and detection method for the application in a Lorentz-force resonance magnetometer based on self-oscillation. The MEMS mechanical structure shows resonance frequency drift with changes in vibration amplitude and the temperature. The presented self-oscillating closed-loop control can reduce the mismatch between drive and resonance frequencies, which causes the large temperature coefficient of the scale factor. Test results indicated that the sensitivity of the proposed device is 59.6 mV/μT and its noise level is 0.25 μT. When operating at ±65 μT, its nonlinearity is 2.5‰—only one-tenth that of the traditional prototype. Its power consumption is about 250 mW, also much lower than that of the traditional prototype. Its size is only 28 mm × 28 mm × 10 mm, that is, about one-eighth the size of the original sensor; further, it can distinguish both positive and negative magnetic fields which is not possible with the former device. The proposed method can also be applied to other MEMS sensors such as gyroscopes and micro mirrors to improve frequency tracking ability. Future research will focus on broadening the bandwidth of the proposed magnetic sensor.

## Figures and Tables

**Table 1:** Comparison of magnetic field sensing system performance.

Author	Dimensions/μm	Bandwidth/Hz	Resolution/nT·mA/√Hz	Current/mA
Kadar [30]	2800 × 1400	N.A.	217	10
Emmerich [31]	1300 × 500	1–10	186	1
Kyynäräinen	2000 × 400	2	7	0.1
Thompson [32,33]	2000 × 1000	1	87	2.7
Alandry [34]	370 × 330	10	1161	4.5
Li [35]	2000 × 2000	1.9	95	8.2
Langfelder [36]	868 × 89	160	520	0.05
Lara-Castro	700 × 600	N.A.	700	20
This study	3000 × 2000	0.3	130	0.4

**Table 2:** Main parameters of MEMS magnetometer structure.

Component	Parameter	Component	Parameter
Pendulum	3000 μm × 2000 μm × 60 μm	Coil layers	2
Hole	30 μm × 30 μm	Coil turns	10
Hole number	864	Coil width	30 μm
Beam length	1350 μm ^1^	Coil thickness	2 μm
Beam thickness	60 μm	Capacitor plate	2300 μm × 1490 μm × 0.3 μm
Beam width	30 μm(Type I) ^1^ 28 μm (Type II) ^1^	Distance between upper and lower plates	10 μm

^1^ Beam length and beam width are equivalent values of the length and width of the fold beam.
